# A Comparison of Innovative App-Based Prescriptions With Conventional Prescriptions for Children by General Dentists: A Mixed Methods Study

**DOI:** 10.7759/cureus.33583

**Published:** 2023-01-10

**Authors:** Shamika Kamath, Ashwin M Jawdekar

**Affiliations:** 1 Dentistry, National Modern (NAMO) Medical Education and Research Institute (MERI) Dadra and Nagar Haveli (DNH), Silvassa, IND; 2 Pediatric and Preventive Dentistry, Bharati Vidyapeeth (Deemed to be University) Dental College and Hospital, Navi Mumbai, IND

**Keywords:** pediatric dentistry, teledentistry, health, telemedicine, dental, pediatric, app, drug dosage, prescription

## Abstract

Introduction

Writing drug prescriptions for children with accurate drug dosages and clear instructions is a must for general dentists. A digital tool in the form of a software application (app) to write, save and share prescriptions can potentially overcome the possible limitations of handwritten prescriptions such as handwriting illegibility, errors in calculations, and incomplete descriptions. However, it is also important to assess the satisfaction of dentists with making prescriptions using a digital tool such as an android application. A mixed methods study comparing an innovative “app-based” and handwritten prescriptions in dental settings is presented.

Methodology

An indigenously developed and piloted app “PREscribing children made EASY (PREASY)” was used in this study. Based on the preliminary study, a sample size of 20 was found to be adequate. Twenty-two dentists participated in the study. The conventional handwritten and an Android app PREASY-based prescriptions were compared in terms of the time taken, precision of writing, and satisfaction of the dentists. Qualitative feedback regarding the PREASY app was obtained in Google Forms (Google, Inc., Mountain View, CA, USA).

Results

A study sample of 20 subjects was calculated based on a pilot study, and 22 dentists were recruited. The mean time taken in seconds for handwritten prescriptions (199.14 (+ 59.18)) was almost four times higher than that for app-based prescriptions (52.05 (+ 23.89)) (t-test, P < 0.00001). The accuracy of handwritten prescriptions versus app-based prescriptions was compared by two examiners independently using analysis of variance (ANOVA) in three domains: dosage accuracy, legible handwriting, and completeness of instructions, the differences (Domain 1 mean + SD: 1.04 + 0.89, Domain 2 mean + SD: 1.38 + 0.40, Domain 3 mean + SD: 0.88 + 0.58) being statistically significant (P < 0.05). The differences in the percentages of the three domains (dosage accuracy: 40.9%, legibility of handwriting: 63.6%, completeness of instructions: 18.1%) were found to be statistically significant (P < 0.05; chi-squared statistic, 9.4017). Of the participants, 59% were very satisfied, 36.3% were satisfied, and 4.5% were neutral (chi-squared test, P < 0.00001). Participants’ feedback/comments were categorized under technical suggestions, dosage suggestions, criticism, and positive remarks and were thematically analyzed.

Conclusion

App-based prescriptions proved to be more instantaneous and detailed than handwritten ones with the majority of dentists satisfied. Valuable feedback pertaining to the limitations of the tool was obtained for improving the app. PREASY-based prescriptions could be recommended for prescribing to pediatric dental patients.

## Introduction

Pharmacotherapy remains one of the major interventional strategies in medicine [1]. Drug therapy in the form of a prescription is the main tool that doctors have for influencing the health of their patients. Prescription writing is an art and science that needs to be mastered by a healthcare professional [2]. Handwritten prescriptions are a norm in dental practice. Digital technology makes it possible to devise application (app)-based prescriptions. PREscribing children made EASY (PREASY) is an indigenously developed Android app for creating prescriptions for pediatric patients in the field of dentistry. There have been no reports of comparative studies of app-based versus handwritten prescriptions by general dentists.

## Materials and methods

Ethical approval

This study was reviewed and approved by the Institutional Ethics Committee of Bharati Vidyapeeth (Deemed to be University) Dental College and Hospital (letter number IEC311022021 dated 03/05/2021). All participants were given an information sheet, and written informed consent was obtained.

Development and workflow of the app

Our technical team developed the PREscribing children made EASY (PREASY) app, which uses the Android platform as it is the most commonly used one in India. The app requires the user/practitioner to log in through a Google account and enter his/her registration number (first screen). The next screen mentions a disclaimer and asks the user to accept the terms and conditions. The third screen allows entering the patient details (patient’s name, age, gender, and weight), after which the subsequent screen appears for the selection of drugs (formulations), including commonly prescribed antibiotics and analgesics to choose from. On ticking the boxes of drugs as per the choice of the dentist, a text message is available on the next screen to view, edit if required, and share the prescription. This app has default settings for calculating drug dosages and selecting the formulation (syrup, tablet, or capsule) and specific instructions pertaining to the selected drug administration. A practitioner may wish to modify any drug or instruction. The YouTube link for understanding the workflow is https://youtu.be/Ewlclukblcs.

Field testing of the app

A pilot study using the prototype of the app was conducted among the postgraduate students in Pediatric Dentistry at Bharati Vidyapeeth University and Maharashtra University of Health Sciences to understand the feasibility of the study and improvements made in the app according to the qualitative feedback received from the participants. The format and contents of the app were validated by the pediatric dentists (authors of the study) and two other dentists. The app including the name (PREASY) and the design is copyright protected.

Study design and data collection

Based on the preliminary study results, this study was conducted among 22 dental practitioners working in different departments of Bharati Vidyapeeth Dental College and Hospital in Navi Mumbai. Dentists practicing general dentistry and not pediatric specialty practice were considered eligible for this study. The study participants were well-informed, and written informed consent was obtained. All participants received training on prescription writing by the same trainer (AJ) with the help of video recording. The clinical scenarios required for writing a prescription were put as chits in a bowl. The participants were told to pick one chit and write a prescription for that particular clinical scenario. Later, he/she was told to make an app-based prescription using the PREASY app. The time taken to write and make an app-based prescription was noted in seconds. Handwritten prescriptions were assessed by two examiners independently for accuracy under the three domains of drug dosage, legible handwriting, and completeness of description of each drug. A few open-ended questions were included in the feedback form (Google Forms, Google, Inc., Mountain View, CA, USA), which the participant was requested to answer on the same day. The study also included a qualitative component.

Descriptive data were exported to Microsoft Office Excel (Microsoft Corp., Redmond, WA, USA) spreadsheet, checked for discrepancies, and processed for inferential statistics.

Statistical analysis

All data were entered into a Microsoft Office Excel spreadsheet and checked for errors and discrepancies. Data analysis was done using Social Science Statistics (https://www.socscistatistics.com/). Parametric tests were used after checking the normality of data using the Shapiro-Wilk z-test. All tests were performed using two-sided tests with alpha 0.05. The results based on descriptive and inferential statistics are presented.

## Results

The sample comprised 22 participants with equal gender distribution. The mean age of the participant was 39.36 (+ 7.52). The mean number of years in practice was 12.59 (+ 6.45). The mean number of children seen per month was 10.22 (+ 11.07). About 23% of the dentists had Bachelor of Dental Surgery (BDS) qualifications, whereas 77% of the dentists had Master of Dental Surgery (MDS) or other postgraduate qualifications. Of the dentists, 66% had single-chair practices, whereas the remaining dentists had multi-chair practices. Descriptive data are presented in Table 1.

**Table 1:** Characteristics of the study population (N = 22) SD: standard deviation, BDS: Bachelor of Dental Surgery, MDS: Master of Dental Surgery

Parameter	Descriptive statistics
Age (mean (+ SD))	39.36 (+ 7.52)
Gender: male	50%
Gender: female	50%
Number of patients seen per month (mean (+ SD))	12.59
Qualification: BDS	23%
Qualification: MDS	77%
Number of years in clinical practice (mean (+ SD))	12.59 (+ 6.45)
Number of patients (less than 18 years old) seen per month (mean (+ SD))	10.22 (+ 11.07)
Type of clinical practice: single chair	66.7%
Type of clinical practice: multi-chair	33.3%

The normality of data was assessed using the Shapiro-Wilk z-test for normality. Data were found to be normal; hence, parametric tests of significance were used (Table 2).

**Table 2:** Shapiro-Wilk z-test for normality

Parameter	Value
P value	0.00007155
W	0.8583
Sample size (N)	44
Sample standard deviation (S)	86.743
Skewness shape	Potentially symmetrical

The mean time taken (in seconds) to complete the handwritten prescription was 199.13 + 59.18, whereas that for app-based prescription was 52.04 + 23.89; the difference was compared using the t-test and found statistically significant (P < 0.00001) (Table 3).

**Table 3:** Comparison of time taken for handwritten prescription versus app-based prescription N = 22 *Significant

Time taken to write a prescription (in seconds)	Time taken to make an app-based prescription (in seconds)	t value = 10.8090	P value < 0.00001*
134	41		
229	42		
172	50		
232	55		
303	43		
289	41		
210	43		
276	112		
227	130		
136	28		
128	40		
167	60		
138	45		
184	66		
97	42		
152	38		
270	38		
247	48		
_250_	50		
197	39		
209	51		
134	43		
Mean = 199.13	Mean = 52.04		
Standard deviation, S: 59.18	Standard deviation, S: 23.89		

The accuracy score for handwritten prescriptions was compared in three domains using analysis of variance (ANOVA); the mean for Domain 1 (dosage accuracy) was 1.04, for Domain 2 (legible handwriting) was 1.38, and for Domain 3 (completeness of instructions) was 0.88. The differences in the mean were statistically significant (P = 0.0386) (Table 4).

**Table 4:** Accuracy score for handwritten prescriptions (ANOVA) N = 22 #Significant at P < 0.05 ANOVA: analysis of variance

Domain 1 (dosage accuracy)	Domain 2 (legibility of handwriting)	Domain 3 (completeness of instructions)	F-ratio = 3.4276	P value = 0.0386*
2	1	1		
0	2	0.5		
2	1.5	1		
2	2	1		
2	2	1.5		
2	1.5	2		
2	1.5	1.5		
0	1	0		
1	1.5	1		
1	1.5	0.5		
1	1.5	1		
2	1.5	1		
0	1	1		
0	1.5	1		
1	1	1		
0	1.5	0		
2	2	1		
0	1	0		
0	1	1		
2	1.5	1.5		
1	0.5	0		
0	1	1		
Mean = 1.04	Mean = 1.38	Mean = 0.88		

The accuracy score for handwritten prescriptions was also compared in three domains using chi-squared statistics; the percent dosage accuracy was 40.9, the percent legible handwriting was 63.6, and the percent completeness of instructions was 18.18 (P = 0.009) (Table 5).

**Table 5:** Accuracy score for handwritten prescriptions (chi-squared test) *Significant at P < 0.05

Parameter	Yes	No	Chi-squared statistic	P value
Dosage accuracy	9	13	9.4017	0.009088*
Legibility of handwriting	14	8		
Completeness of instructions	4	18		

Among the participants, 59.09% were very satisfied, 36.36% were satisfied, and 4.54% were neutral. The chi-squared statistical comparison showed significance (P = 0.0006) (Table 6).

**Table 6:** Satisfaction for app-based prescriptions by the participants N = 22 *Significant at P < 0.05

Response category	Number of responses	Percentage	Chi-squared statistics	P value
Very satisfied	13	59.09	14.8636	0.000592*
Satisfied	8	36.36		
Neutral	1	4.54		
Dissatisfied	0	0		
Very dissatisfied	0	0		

Participants’ qualitative feedback for the app-based prescription was thematically analyzed. Four predominant themes emerged: technical suggestions, dosage suggestions, criticism, positive remarks, and any other comments (Table 7).

**Table 7:** Thematic analysis of participants’ qualitative feedback for the app-based prescription

Theme	Analysis
Technical suggestions	The safety and privacy of data should be maintained. There should be ease of incorporation of and editing of letterhead and logo. Let the prescription be shared in PDF format only. WhatsApp text can be edited. Let the prescription have the app watermark. In the instructions, a note can be added that the patient should acknowledge after getting the prescription to ensure the prescription has come through the proper channel. Add the patient’s address too. Adult prescription data should be included. Kindly make it available on iOS as well. Registration should be limited to practitioners only. PDF format should be sent to WhatsApp as no one can tamper with it. More medications can be included so that the app can be used by pediatricians too.
Dosage suggestions	Automatic. Looks good. Should have options to choose tablet/syrup/syrup of different drug strengths.
Criticism	Only available on Android and not on iOS.
Positive remarks	Great efforts. Congratulations. It’s a good app and saves time. Will make prescriptions easier. It’s very helpful in busy clinical practice, and it’s a very time-saving app, so very glad to use it. Very easy to use and saves a lot of time. Very nice thought and made with accuracy, had a great experience. Easy and faster to use. Very informative. Convenient. Very good. Lovely app. It’s a time-saving app. Very thoughtful and time-saving app. Very good, very innovative. Good innovation; it makes prescription easier. Useful. Good potential. Outstanding. Accurate and time-saving. Helpful for new practitioners. Very easy to use.
Any other comments	Hope it can be replicated for adult patients. The initial data feed can also include “allergies to any medicine.” Element of bias as the participant is already sensitized while filling the written prescription, hence filling on the app is faster. Suggestions would be to ask each participant to fill four prescriptions - two will be written first and the other two will be app first and then take mean of the readings. Good app for general dentists.

## Discussion

Prescribing is an indispensable skill for doctors in all medical specialties. Writing a prescription is integral to the practice of dentistry [3]. Prescription is written information or any advice provided for patients that bring into focus the diagnostic acumen and therapeutic proficiency of a physician. It also includes instructions for the restoration of a patient’s health [4]. The World Health Organization (WHO) recommends that a prescription should identify the professional, patient, mode of administration, pharmaceutical form of the medicines, dosage, frequency of use, and duration of treatment, along with patient guidance and information [5]. Extreme caution should be exercised to avoid errors in medical prescriptions as they may not only lead to difficulties and mistakes in dispensing medicines but also result in incorrect drug use that can make treatments ineffective or unsafe [3]. A well-documented prescription is not only a legal necessity but also a practice-builder for the dentist.

Prescribing medicines to children needs precision in terms of calculating drug dosages appropriately [6]. As pediatric dosages are calculated based on the child’s age and weight, the risk of errors in dosage calculations is increasing. Dental professionals have to be very skillful in mathematical calculation and numerical ability while calculating drug dosages for children. On the other hand, illegible handwriting can lead to misinterpretations of dosage, drug name, or abbreviations [7], with subsequent medicolegal implications in addition to errors in drug dispensing and administration.

The dental profession has experienced a dramatic acceleration in the use of communication systems and information-based technologies. Information technology has enabled the digitalization of dental records, and the same can be used for both calculating drug dosages and creating a prescription. The education and training of prescribers and the use of online aids can inculcate the practice of sound prescription writing [8]. Prescribing is an individualized and dynamic clinical process that can be standardized through e-prescribing systems. The role of e-prescribing in mitigating medication errors, improving communication with dispensing pharmacists, and improving medication adherence is crucial in drug therapy. The American Academy of Pediatrics (AAP) recommends the adoption of e-prescribing systems with pediatric functionality [9]. There is evidence that computerized prescribing has improved legibility and clarity, saved time, and reduced the time spent clarifying prescriptions with the dispensing pharmacist [10].

The present era of smartphones has people turning toward mobile-based applications (apps) to achieve daily goals. Healthcare has also witnessed tremendous growth in technology-based healthcare delivery systems. Technological advances have made it possible to digitize records and appointments; however, such technological support for writing a prescription is minimal as of now. In the field of dentistry, there are only a few apps that make prescription writing easy, but to the best of our knowledge, a pediatric drug dosage app is not available to date. A digital tool to write, save, and send prescriptions can potentially overcome the limitations of handwritten prescriptions. Few studies in dental literature have explored software-based prescriptions and compared them with handwritten ones. With this goal in mind, an innovative app-based system, PREASY (an Android app), was developed in an attempt toward encouraging dental practitioners to e-prescribe the correct drug dosage in pediatric patients. This research project included developing PREASY app for the purpose of writing a prescription for pediatric dental patients and testing its use by dental practitioners. Mobile health (mHealth) is a mobile technology that supports mobile healthcare. With mHealth, it has become easier for a user to access web-based applications and websites [11].

In the first part of the project, with postgraduate students as participants, the development of the PREASY app, its feasibility, functionality, and preliminary study (app testing) results were presented in a previous report [12]. In the second part, with general dentists as participants, this study compared the time taken by dental practitioners for handwritten prescriptions and PREASY app-based prescriptions. This mixed methods study also assessed the accuracy of handwritten prescriptions, compared dentist satisfaction toward app-based prescriptions, and analyzed the feedback of dentists to app-based prescriptions qualitatively.

This study also revealed similar results as the preliminary study. The time taken to make an app-based prescription was one-fourth of the time taken to write a prescription, as compared to the preliminary study, in which the time taken to make an app-based prescription was one-third the time taken to write a prescription. This difference could be attributed to the difference in the overall training and experience of participants as this study included general dental practitioners and the preliminary study included postgraduate students. Also, in the three domains of accuracy, the results of this study and the preliminary study were found to be comparable. Moreover, with regard to the satisfaction of the participants, there were no dissatisfied participants.

Although the results of the qualitative study were positive, the themes suggest preferences (e.g., iOS platform) that the dentist would expect from e-prescriptions. There were a lot of technical suggestions regarding privacy and maintaining data and making tamper-proof prescriptions.

This study was conducted in accordance with the guidelines of Good Reporting of A Mixed Methods Study (GRAMMS) for reporting mixed methods studies [13]. A mixed methods study design is paramount for recording both subjective and objective parameters such as time taken, accuracy, satisfaction, and positive and negative criticism.

As with any technological advancement, the PREASY app also has certain limitations. This app is useful only for those using smartphones. The other limitation is that the current app includes drugs that are commonly used, and the clinical scenarios required the use of only those drugs. Furthermore, PREASY is an Android-based app and is currently not available for iOS users. The effect of parameters such as gender, qualification, number of years in practice, and single or multi-chair practice was not assessed because of the small sample size. Despite such limitations, this app lays a foundation for future research on e-prescribing.

## Conclusions

The knowledge of prescribing drugs is of utmost need for good dental clinical practice. The PREASY app seems to be a promising mHealth app and could play an instrumental role in fostering the standardization of drug prescriptions in pediatric dental patients. Although the app was found to be effective in this study, more randomized studies in diverse settings are required to establish the advantage of these app-based prescriptions over handwritten ones.
